# Ruptures of mixed lipid monolayers under tension and supercooling: implications for nanobubbles in plants†

**DOI:** 10.1039/d4na00316k

**Published:** 2024-06-11

**Authors:** Stephen Ingram, Bernhard Reischl, Timo Vesala, Hanna Vehkamäki

**Affiliations:** a Institute for Atmospheric and Earth System Research/Physics, Faculty of Science, University of Helsinki P.O. Box 64 Helsinki FI-00014 Finland stephen.ingram@helsinki.fi; b Institute for Atmospheric and Earth System Research/Forest Sciences, Faculty of Agriculture and Forestry, University of Helsinki P.O. Box 27 Helsinki FI-00014 Finland

## Abstract

Mixed phospholipid and glycolipid monolayers likely coat the surfaces of pressurised gas nanobubbles within the hydraulic systems of plants. The lipid coatings bond to water under negative pressure and are thus stretched out of equilibrium. In this work, we have used molecular dynamics simulations to produce trajectories of a biologically relevant mixed monolayer, pulled at mild negative pressures (−1.5 to −4.5 MPa). Pore formation within the monolayer is observed at both 270 and 310 K, and proceeds as an activated process once the lipid tails fully transition from the two dimensional liquid condensed to liquid expanded phase. Pressure:area isotherms showed reduced surface pressure under slight supercooling (*T* = 270 K) at all observed areas per lipid. Finally, Rayleigh–Plesset simulations were used to predict evolving nanobubble size using the calculated pressure:area isotherms as dynamic surface tensions. We confirm the existence of a second critical radius with respect to runaway growth, above the homogeneous cavitation radius.

## Abstract 2

Mixed monolayers likely coat the surfaces of gas nanobubbles within the hydraulic systems of plants. By calculating their dynamic surface tensions, we predict evolving bubble radii and stability under negative pressure.

## Graphical abstract

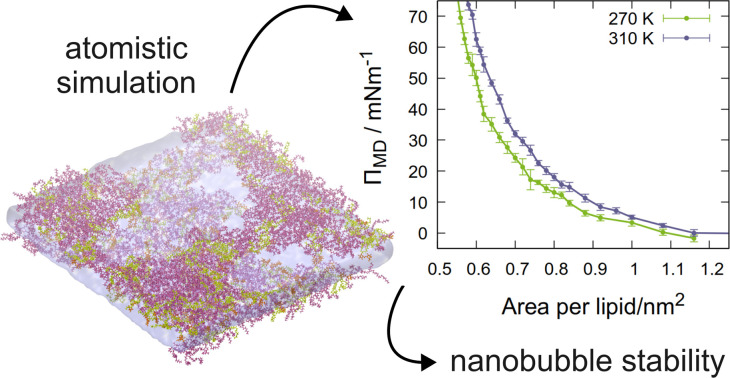

## 1 Introduction

Trees are capable of a remarkable feat of engineering: they can transport water against gravity, non-mechanically, under negative pressure.^1^ An unbroken column of tree sap stretches through porous tissue in the outermost ring of the trunk, connecting the roots, where water is absorbed, to the leaves, where it is released. Consequently, tree sap can exist in a state some have described as “doubly” metastable:^2^ the liquid is transported under tension, and, in some climates, below its freezing point for much of the year.

In the tree science community, negative pressure is referred to as ‘water potential’, *Ψ*_water_ (per the Gibbs–Duhem equation, chemical potential per unit volume has the dimensionality of pressure) and arises from water being stretched away from its equilibrium density.^3,4^ The entirety of the driving force for the stretching, and hence the upward flux of water against gravity, is produced by evaporation within confined channels on the leaf surface called stomata.^1^ The magnitude of the negative pressure within the leaf tissue, Δ*P*_leaf_, can be calculated using the Kelvin equation, by considering the water activity gradient directly above the leaf,^5,6^1
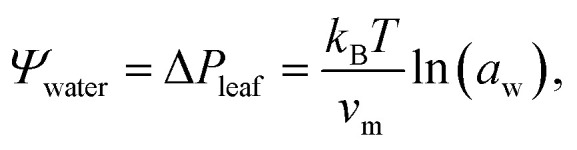
where *k*_B_ is Boltzmann's constant, *T* is the temperature, *v*_m_ is the molecular volume of water, and *a*_w_ is the ambient relative humidity at sufficient distance from the leaf surface. Water potential is therefore the most negative directly below the stomata, and it is only here that the first equality of eqn (1) holds. Further from the leaves, the magnitude of *Ψ*_water_ decreases (becomes less negative) and approaches a value of Δ*P*_root_ at the external membranes of the roots.^1^

**Fig. 1:**
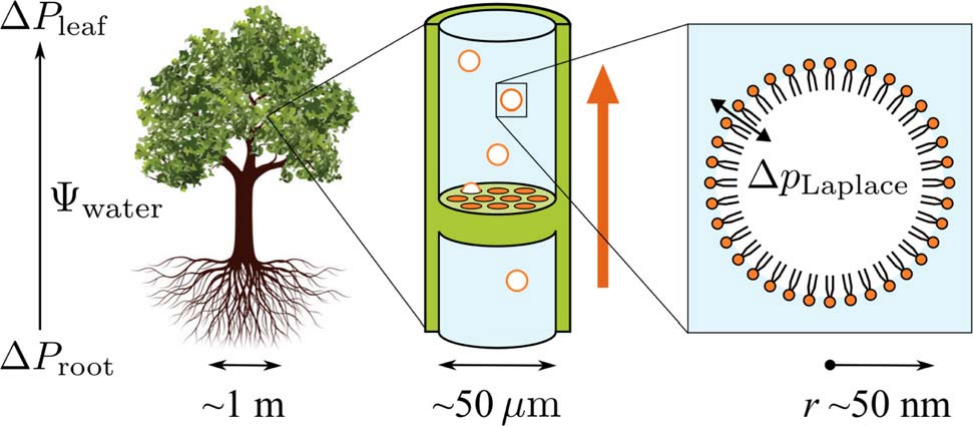
Schematic representation of the tree hydraulic system, showing processes occurring on three length scales: negative pressure causing upward motion of sap through the xylem tissue (left), air seeding of nanobubbles from pit membranes within a xylem conduit (middle), and a single lipid coated nanobubble stabilised by a large Laplace pressure gradient across its interface (right).

As shown in the schematic Fig. 1, sap water moves from root to leaf through rows of microscopic conduits, collectively called xylem. The xylem walls are reinforced with lignified material for rigidity, but also contain so-called pit membranes embedded into them. Pit membranes exhibit a partially porous structure, formed of overlapping lignin fibres^7,8^ which allow lateral (or vertical) movement of water between conduits. Generally, hydraulic conductance can be maintained even when dissolved gases are present, however it is possible for individual conduits to embolise, *i.e.* to drain of water fully and become filled with gas.^9^ If an embolised conduit is adjacent a liquid filled one, the combination of (positive) gas pressure on one side of the pit with liquid under negative pressure on the other will extrude nanobubbles through the porous mesh, and into the bulk liquid.

It has become clear in recent years that pit membranes are coated with large concentrations of lipids, in either monolayer or micellar form.^10^ Lipid composition within the xylem sap water itself has also been studied, *via* mass spectrometry, in several species of tree.^11^ While the ratios of specific lipids varied, phospholipids tended to dominate, with glycolipids and triglycerides appearing at lower concentrations. Any air nanobubbles seeded through a pit membrane will therefore be coated with a monolayer of surfactants during the formation or “budding off” process. These coatings have also been observed within cryo-SEM images of frozen xylem sap samples.^12^

The internal (positive) pressure of gas that can exist inside a nanobubble at equilibrium is determined by the Laplace equation:2
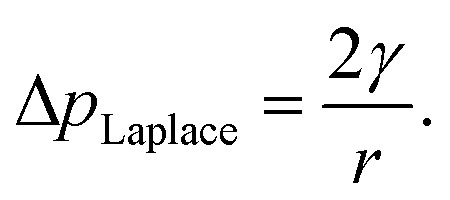


Depending on the surface tension, *γ*, exhibited, eqn (2) predicts that a bubble of radius *r* = 50 nm could sustain a pressure differential across its interface of between 1.8 and 2.7 MPa, corresponding to the observed bulk xylem surface tension range of 45 and 68 mN m^−1^ respectively.^13^ Due to the direction of the applied force, a surrounding water potential of *Ψ*_water_ = −1 MPa would lower, rather than raise, the internal bubble pressure, to 0.8–1.7 MPa. As described above, this effect would become more pronounced closer to the leaves, as the contribution of *Ψ*_water_ to Δ*p*_Laplace_ becomes more significant.

However, these surface tensions are bulk values that do not take into account that the concentrations of lipids at the bubble surface will be significantly higher than within the xylem sap as a whole. Furthermore, *γ* itself is not static with respect to either radius or the physical conditions the bubble experiences. It must be represented by a full pressure area isotherm, dependent on both *Ψ*_water_ and temperature.

Majewski *et al.*^14^ found that doping a phosphatidylcholine (DPPC) monolayer with up to 20 mole% of the glycolipid GM1 had little impact on the pressure:area isotherms at a temperature of 23 °C, despite monolayers of the pure glycolipid having a much broader and shallower increase in surface pressure during compression. The same system was investigated by Frey *et al.*^15^ with a larger range of mixing ratios, who found that small amounts of glycolipid have a condensing effect on the monolayers, and only deviate significantly from the pure DPPC dependence at an above 50 mole% GM1. Unfortunately, neither of these lipids occur in tree sap, nor do these studies offer insight into the behavior of monolayers under supercooled or negative pressure conditions.

To our knowledge, there has been no investigation into the effect of temperature on mixed monolayers containing glycolipids and phospholipids. In our previous work,^16^ we calculated dynamic surface tensions of a prototypical glycolipid as a function of applied negative pressure. A kinetic effect was observed where larger pulling rates reduced the surface pressure, *Π*_MD_, as the interface expanded faster than the lateral diffusion of the lipids could compensate. Conversely, a monolayer comprised of a prototypical phospholipid was found to rapidly condense into a stable lamellar-like liquid condensed (LC) phase, rendering it highly resistant to pulling, even for pressures as negative as −3.5 MPa.

These results emphasize that the instantaneous surface tension of a nanobubble's coating determines not only the pressure differential it can sustain, but also its stability with respect to embolising the conduit it is in. In this work, we wish to investigate the temperature dependence of dynamic surface tension in a two component monolayer, under a large (positive) gas pressure. Results will then be parameterised into a Rayleigh Plesset model^17,18^ as dynamic surface tensions as a function of bubble radius, *γ*(*r*). We will show that such a model can be used to predict the regions of the [*p*, *T*] parameter space in which bubbles remain stable in size, and those in which they expand spontaneously into embolisms, where *p* is the negative liquid pressure acting on the bubble's surface.

## 2 Computational methods

We have utilised molecular dynamics simulations to investigate mixed monolayers of phospho- and glycolipids, using the CHARMM36 biomolecular force field. Plant lipids generally possess polyunsaturated tail groups,^19^ and therefore exhibit low liquid condensed/liquid expanded (LC/LE) transition temperatures. In recognition of this, we have specifically used dilinoleoyl-phosphatidylethanolamine (DLiPE 18 : 2/18 : 2) and digalactosyl-diacylglycerol (DGDG 18 : 3/16 : 3) in this study. These lipids have been found to exhibit LC/LE transition temperatures (under positive lateral pressure) of 258 and 233 K respectively, suggesting they will be primarily in the LE phase at the temperatures studied here.^20,21^ To avoid confusion with dilauroyl-phosphatidylethanolamine (DLPE) the phospholipid will be referred to hereinafter by the more general term PE, as this is how it is named in the tree science community. We have represented the PE head group as zwitterionic as, at the close to neutral pH of xylem sap, phosphate groups will be deprotonated and ammonium groups will be protonated.^12,22^

**Fig. 2:**
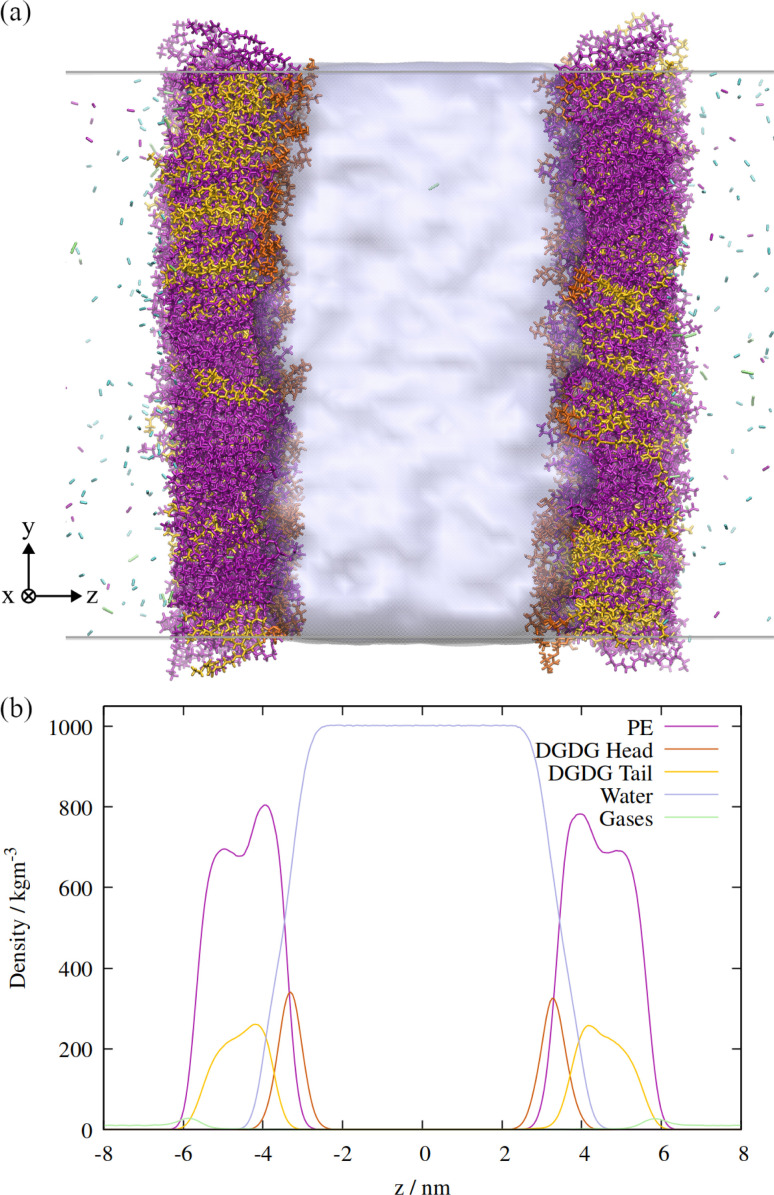
(a) Side view of the atomistic model of the double interface of a gas bubble–lipid monolayer–water system, investigated at an area of 0.55 nm^2^ per lipid. Lipid and gas molecules are shown in licorice representation with PE in purple, DGDG in orange (head) and yellow (tail). The surface of the water volume is shown in light blue. Note that the two gas volumes on either end of the simulation box are connected through periodic boundary conditions and constitute the “inside” of the bubble, whereas the water volume in the center of the box represents the liquid surrounding the bubble. (b) Associated density profiles of each component, calculated as a function of *z* coordinate, averaged over a 60 ns molecular dynamics trajectory. Water and lipid molecules are colored as in panel (a).

An orthographic projection of the system viewed along the *x* dimension is shown in Fig. 2, along with the associated lateral densities of the components after equilibration. The mixing ratio was 3 : 1 PE : DGDG by number and there were 300 lipids in each monolayer. Such a ratio reflects the general trend in sap concentrations of the two lipid types, although compositions vary substantially from species to species.^10^

The Kirkwood–Buff method of calculating surface tension from molecular dynamics simulation,3
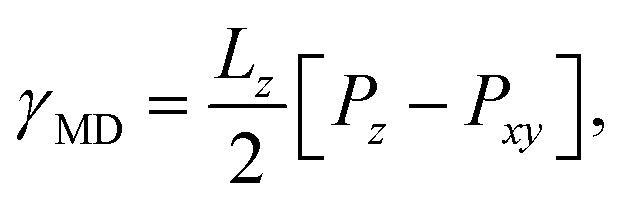
describes that the presence of an interface between two phases will induce a difference in pressure between the in plane (*P*_*xy*_) and out of plane (*P*_*z*_) dimensions of the surface. In atomistic simulations, there are usually two interfaces present: the top and bottom of a liquid slab, placed within a box of vertical size *L*_*z*_. Note that the above equation was derived from consideration of a system in which the mean distance between interfaces does not change over the timescale probed.^23^ Therefore, constant volume simulations containing high Laplace pressures of gas in contact with the lipid interfaces are most likely to give accurate results in this context.^24^

Two monolayers of 300 lipids were represented by the CHARMM36 force field, and the initial configurations generated using the CHARMM-GUI interface,^25^ with initial areas per lipid specified as 50 Å^2^ for PE and 75 Å^2^ for DGDG. The number of water molecules within the slab was specified to be approximately 60 per lipid, and a total of 38 711 molecules were generated. These were initially generated as TIP3P molecules, which were then converted to the TIP4P/Ice model^26^ by adding an additional dummy atom at the same location as the second hydrogen atom of each molecule. As the dummy atom had no mass, it did not ‘collide’ with the H atom and so moved to the correct position when a steepest descents minimization was applied with the appropriate water model topology. The resultant monolayer configurations were then equilibrated in the NpT ensemble with a positive lateral pressure of 1 atm and *T* = 290 K, according to the generated CHARMM-GUI MD parameters, *i.e.* using distance restraints that loosened progressively over six steps.

To produce the input coordinates for the pulling simulations, the gas phase was then populated with molecules using the GROMACS insert-molecules command. Van der Waals radii were scaled to be twice their initial value to prevent gas molecules being inserted inside the monolayer or the water slab. The composition was calculated using the corrected ideal gas law for each species, with target pressures of 0.8 MPa N_2_, 0.1 MPa O_2_ and 0.1 MPa CO_2_. The numbers of molecules are as follows: 766 N_2_, 96 O_2_ and 96 CO_2_. CO_2_ was represented by the flexible angle EPM2 model^27^ and N_2_ and O_2_ by the rigid models described by Vujić and Lyubartsev.^28^

NpT ensemble pulling simulations were each 20 ns in length and NVT ensemble surface tension simulations were each 60 ns. They were performed on the Puhti cluster operated by CSC, utilizing a configuration of 32 CPU cores and a single Nvidia v100 GPU. The GROMACS version was 2021.5.^29^ The Nosé–Hoover expanded ensemble thermostat^30^ was used for all pulling and ‘production’ isotherm simulations. Solvent, monolayer and gas groups were coupled to separate heat baths, each with a time constant of 0.75 ps. The Parrinello–Rahman barostat^31^ was used for the pulling simulations, with a time constant of 5 ps and a compressibility in the *xy* plane of 5 × 10^−6^ bar^−1^, per Duncan and Larson.^32^ The compressibility in the *z* dimension was set to zero in all pulling simulations, meaning that only the lateral area was changing. The Verlet list, van der Waals and coulombic cut-offs were all set to 1.2 nm, with a switching radius of 1.0 nm, per the CHARMM-GUI recommendations. Long range electrostatics were calculated using the particle mesh Ewald method, with a Fourier spacing of 0.175 nm. Long range dispersion corrections were applied to both the energy and pressure. The LINCS algorithm^33^ was used to constrain all bonds containing hydrogen atoms, with an order of 4 in the coupling matrix expansion, and a single iteration in the final step.

Surface tensions were converted to surface pressures according to the equation

4 *Π*_MD_(*T*) = *γ*_water_(*T*) − *γ*_MD_(*T*),

where the temperature dependent pure TIP4P/Ice surface tensions, *γ*_water_(*T*), were calculated from simulations identical to those described above, but with the lipids removed from the interface. The calculated values were 76.3 ± 0.7 mN m^−1^ at *T* = 270 K and 70.5 ± 1.1 mN m^−1^ at *T* = 310 K.

In order to assess the local phase environment of the lipid tail groups, and track pore formation within the monolayer, Voronoi tessellations were produced using the Voronoi library within the SciPy python module. The mdtraj module^34^ was used to load the trajectories and select the appropriate atoms, with position data extracted for both monolayers at time increments of once per 2.5 ns, with the first 10 ns excluded. We will briefly describe the procedure, which was applied to both monolayers separately, with the results then averaged:

First, an initial Voronoi tessellation was calculated for the *xy* coordinates of the sixth carbon atom within each tail group. To avoid the tail group atoms at the edge of the simulation box being classified as having fewer neighbors than in actuality, it was necessary to then reconstruct the atoms on the other side of the periodic boundary. This was achieved by identifying any tail group atoms whose polyhedra contained vertices outside the periodic box. The *xy* positions of these atoms were then translated by one box-width in both dimensions, and were then appended to the input coordinates, at which point the Voronoi tessellation was recalculated.

Once this was complete, tail group atoms were classified into the appropriate two dimensional phase: liquid condensed was defined as having six or more neighbors within a radial distance of 0.8 nm, gas (*i.e.* at the pore edge) if at least one neighbour was over 1.5 nm away, and in the liquid expanded phase otherwise. The pore radii were calculated separately using the largest distance between a series of grid points and their nearest atom in the *xy* plane, using the same atoms as were used to calculate the Voronoi tessellations. A similar procedure has been described in three dimensions for cavitation simulations by Gonzalez *et al.*,^35^ and the exact procedure was reported in our previous publication.^16^ A grid size of 40 × 40 was used. All error bars presented in this paper, for surface tension, LC/LE ratios and pore radii, represent the standard deviations of the calculated values between *t* = 10 and 60 ns.

The time evolution of three dimensional bubble sizes subject to dynamic surface tensions were calculated by integrating the modified Rayleigh–Plesset equation (7). The variable coefficient ODE solver provided by SciPy was used, with the backward differential formula method and a timestep of 10 ps. *γ*(*r*) was represented by fitting a sum of two error functions to the calculated *γ*_MD_(*T*) values, with 
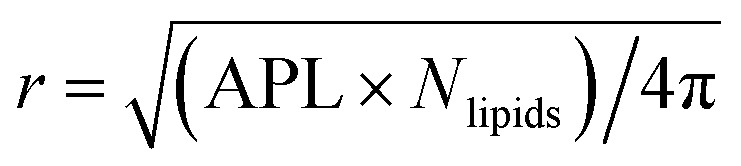
 (see Fig. S4†). The viscosity of water, *η*, was calculated as 2.00 × 10^−3^ Pa s at 270 K and 0.69 × 10^−3^ Pa s at 310 K using the parameterisation of Dehaoui *et al.*^36^ Thermal noise was added as described in Menzl *et al.*,^37^ according to the formula 
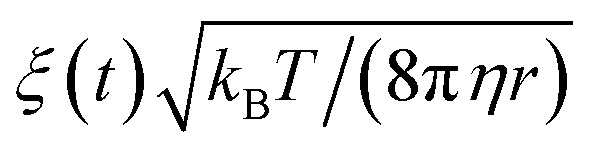
, where *ξ*(*t*) samples from a Gaussian distribution with a unit standard deviation, centered at zero.

## 3 Results and discussion

First, a series of lateral pulling trajectories were conducted in the NpT ensemble, at *T* = 270 K and 310 K. We believe these to be reasonable upper and lower limits for the temperatures experienced by the hydraulic systems of most vascular plants. 270 K is within the range of supercooled temperatures that sap present in xylem conduits can withstand before freezing.^2^

**Fig. 3:**
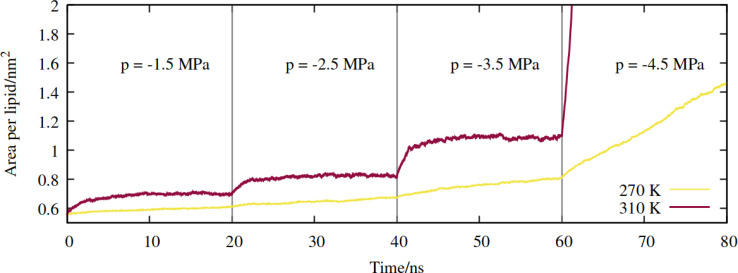
Expansion of the mixed monolayer during sequential pulling simulations, with increasingly negative pressure *p*, at *T* = 270 K (yellow) and 310 K (red).

Increasingly negative pressures were applied in the *xy* plane, in increments of 20 ns, to induce expansion of the mean area per lipid (APL). In contrast to the pure phospholipid system investigated in our previous work, expansion of the mixed monolayer occurred relatively rapidly at both temperatures studied. More extreme pulling rates induced faster expansion and the system plateaued at a more stretched configuration relative to the starting coordinates. Fig. 3 shows the 270 K system adopting larger equilibrium APL values during the first three NpT simulations, corresponding to pressures of −1.5 to −4.5 MPa, in increments of 1.0 MPa.

The 310 K system exhibited similar dynamics within the first three simulations, albeit with slightly larger equilibrium areas per lipid and a more pronounced expansion within the first few ns after the pressure was increased. During the −4.5 MPa simulation the monolayer ruptured and the slab underwent runaway growth. It is clear that the monolayers were close to a critical configuration with respect to rupture at *T* = 310 K and *p* = −3.5 MPa, while the supercooled system adopted a lower area per lipid at the same pressure, and expanded more slowly. To assess the effect of simulation length, and to probe the longer timescale response of the monolayer to pulling at *T* = 270 K, we continued the *p* = −2.5 MPa trajectory up to 200 ns. The APL values plateaued at 0.73 nm^2^ after 80 ns, and fluctuated around that value for the rest of the trajectory (Fig. S1†); the dynamics were approximately a factor of 20 slower than at *T* = 310 K, for a smaller total increase in simulation box area.

Therefore, somewhat counter-intuitively, the monolayer in the doubly metastable state (negative pressure + supercooling) was more stable with respect to rupture than in the singly metastable state. The explanation for this is as follows: since pore formation is an activated process in self assembled lipid nanostructures,^38,39^ it requires both thermal energy and pulling to occur at high probability within a single MD trajectory. A deficit in one (a lower temperature) must be made up for by the other (a more negative pressure).

### 3.1 Temperature dependent surface tension

Directly calculating the surface tension of the evolving monolayer as a function of area within NpT simulations is undesirable because, as described earlier, the interlayer distance varies over time. Therefore, to produce pressure:area isotherms across the APL range traced by the above trajectories, a total of 49 frames were selected, between 0.55 and 1.16 nm^2^ per lipid (24 frames for 270 K, 25 frames for 310 K). Each area was then simulated in the NVT ensemble, as described in Section 2, with the *x*, *y* and *z* components of the pressure tensor output every 2 ps. The first 10 ns was removed from the analysis, to allow the newly randomized velocities to decorrelate from their initial states. In addition, a final frame was extracted from the NpT trajectory of the 310 K system, in the fully ruptured state of 2.00 nm^2^ per lipid, which will be discussed in Section 3.2.

The APL = 0.55 nm^2^ per lipid configuration was considered close to the equilibrium density at 270 K as, by virtue of the compression steps during equilibration, it was the only frame for which the lateral components of the pressure tensor were positive. Conversely, at 310 K, the calculated surface tensions *γ*_MD_ were themselves negative at APL < 0.59 nm^2^. This suggested that 0.59 nm^2^ per lipid was the closest to a completely stabilised interface that could be sustained under conditions of constant pulling at this temperature, and that smaller APL configurations would be overly condensed. The trend in calculated surface tensions bears this assumption out.

**Fig. 4:**
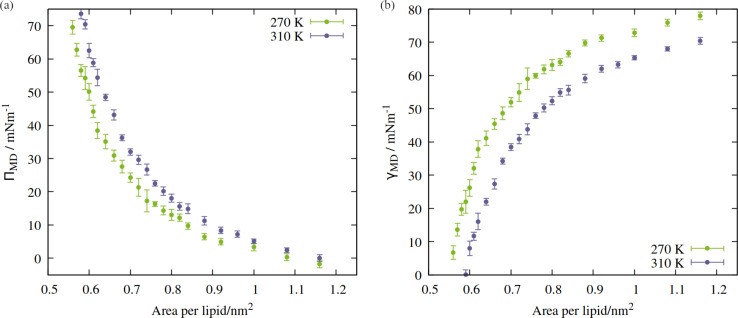
Isotherms showing (a) surface pressure, and (b) surface tension as a function of the area per lipid within the studied mixed monolayer at temperatures *T* = 270 K (green) and 310 K (blue). Error bars represent standard deviations between *t* = 10 and 60 ns.

The shape of the pressure:area isotherms (Fig. 4, panel a) are as expected between 0.55 and 1.16 nm^2^ per lipid, namely they show a collapse towards zero as the expansion proceeds and the outward force exerted by the monolayer on the periodic boundary decreases. Within this area range, the surface tension *γ*_MD_ was larger at 270 K than 310 K and the surface pressure *Π*_MD_ was lower: the thermal energy of the system was smaller relative to the surface free energy, meaning that the energetic cost of expanding the area was increased, by at least 10 mN m^−1^, in the approximate range 0.6 < APL < 0.75 nm^2^. At higher areas, the two *Π*_MD_ values become closer to one other, as they both tend to zero.

According to eqn (2), a larger surface tension at the critical area corresponds to a more negative Δ*p*_Laplace_ that can be sustained across the interface without rupturing. To draw an analogy with the plant hydraulic system, bubble surfaces resembling those investigated here will be more stable with respect to embolism at lower temperatures, both at a given xylem water potential, *Ψ*_water_, and across a wider range. This result is consistent with some previous tree level experiments; Cochard *et al.*^40^ produced negative pressures in cut branches using a centrifuge, at temperatures between 50 and 1 °C. They found that cooling the branches made them more resistant to embolism at a given negative pressure.

However, it is difficult to generalise from experiments where *p* and *T* are varied independently: they are correlated within the plant hydraulic system and change in a complex and path dependent manner. For example, increased temperatures can reduce soil water content, stressing plants and generating more negative pressures within them. Other environmental factors can affect *Ψ*_water_ on both a diurnal and annual timescale, such as ambient relative humidity, per eqn (1), or the rate of photosynthesis throughout the day. These feedback loops make it difficult to infer a causal relationship between quantities like surface tension and the macroscopic rate of embolism.

### 3.2 Lipid phase characteristics

**Fig. 5:**
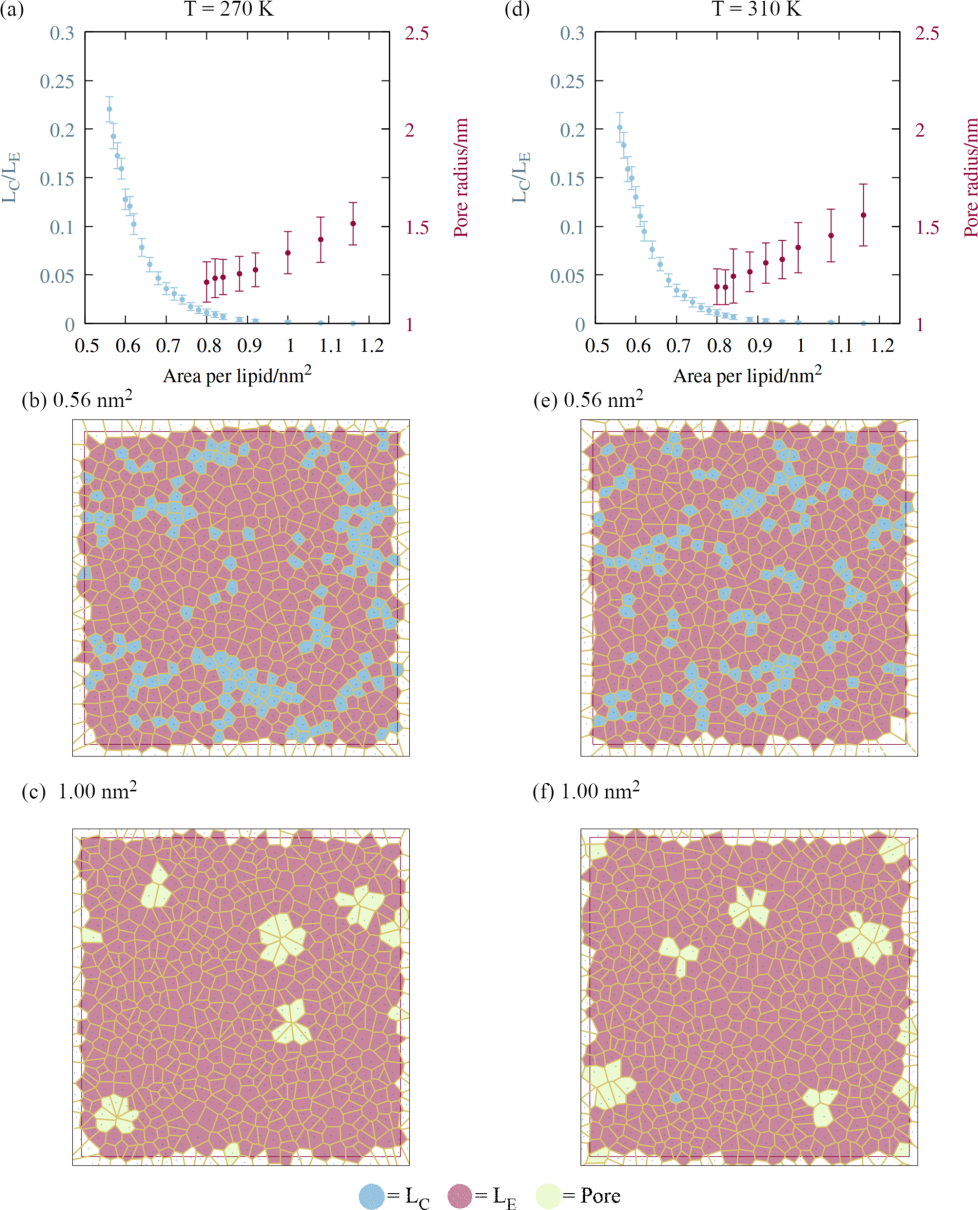
The impact of monolayer expansion on the phase state of the lipid tails at the two temperatures studied. (a) Reduction in the amount of liquid condensed phase (blue points) and increase in mean pore size (red points) at *T* = 270 K. Error bars represent standard deviations between *t* = 10 and 60 ns. Voronoi tessellations of lipid tail atoms at *t* = 60 ns are presented for areas per lipid of (b) 0.56 and (c) 1.00 nm^2^, colored according to phase state. Panels (d)–(f) follow (a)–(c) for *T* = 310 K.

A script was written to classify the two dimensional phases of the monolayer lipids using a Voronoi tessellation of the sixth atoms in each of the lipid chains, as described in Section 2. The proportion of atoms in the liquid condensed phase across the APL range is shown in Fig. 5, panels a and d. Once the monolayers began to rupture, the largest pore radius was also calculated for the same frames. As expected, a sequential coexistence of phases is observed as the monolayer is pulled apart: the initial proportion of lipids in the LC phase at an area per lipid of 0.56 nm^2^ was 22 ± 1.3%. During subsequent pulling, the LC–LE coexistence region monotonically becomes an entirely LE leaflet, which then ruptures at further expansion, becoming LE–gas (or LE + pore).^24^ The lipids reorganise cooperatively, and move laterally through the leaflet at the same rate, although DGDG tail atoms were rarely found in the LC phase. This is likely a consequence of the additional double bond in both tail groups reducing packing efficiency.

The lower contact between the tail groups at higher APL values (1.00 nm^2^) can also be seen in the presence of several ∼1.5 nm radius pores in the Voronoi tessellations (panels c and f). These open and close stochastically as the angle of the tail groups fluctuates with respect to the interface, suggesting they are ‘subcritical’ pores. Analysis of the radial distribution functions (Fig. S2†) reveals that the correlation within the first solvation shell becomes smaller as the interfacial area increases, indicative of weakening intermolecular bonds relative to the thermal energy *k*_B_*T*. Peaks corresponding to solvation shells beyond the third disappear at APL = 1.08 nm^2^, confirming the loss of long range order.

Pore formation in a monolayer can be thought of as a cavitation process occurring in two dimensions.^38^ Therefore, a monolayer in the fully LE phase state can be made metastable with respect to the nucleation of a stable pore by further pulling under negative pressure. We note that (1) the transition of all tail groups into the LE phase, (2) the initial observation of subcritical pores, and (3) the collapse of the surface pressure to zero, all occur at approximately the same APL. From an energetic standpoint, (3) is especially important, as it means that the monolayer is no longer acting to reduce the surface free energy from that of pure water. As a result, increasing the area by exposing a direct water–gas interface will not be penalized, relative to further expansion that does not nucleate a pore.

The temperature itself does not appear to affect the process mechanistically, at least within the range studied here. We can say that a bubble's coating will be more condensed, in terms of APL, under supercooling. Additionally, it will likely expand at a slower rate after pore formation has been initiated, as with the ruptures observed in Fig. 3.

**Fig. 6:**
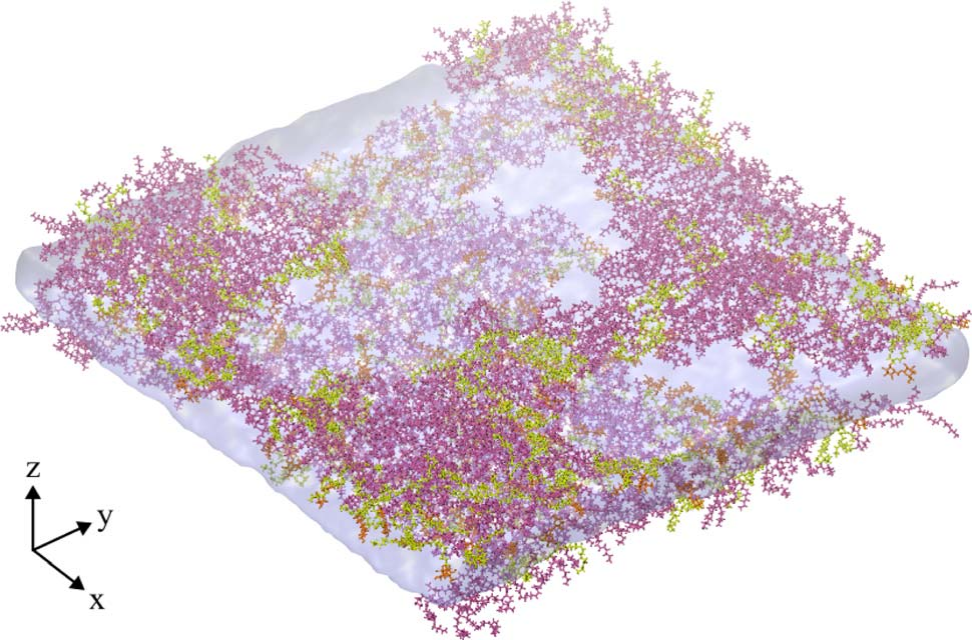
Snapshot of the final frame of an NVT ensemble trajectory conducted at an area per lipid of 2.00 nm^2^, and *T* = 310 K, showing a fully ruptured monolayer system. Water and lipid molecules are colored as in Fig. 2a, gas molecules are not shown.

A final trajectory was conducted at an APL value of 2.00 nm^2^ at *T* = 310 K, during which the monolayers had lost structural integrity, transitioning to a network of interconnected LE leaflets between large pores of relatively stable size (Fig. 6). The mean pore radius was calculated as 7.30 ± 1.03 nm. Density iso-surfaces of the water slab show that the water level within the stable pores is slightly closer to the gas phase than the slab underneath the membrane, *i.e.* it rises to occupy the center of mass of the remaining monolayer.

### 3.3 Rayleigh–Plesset dynamics

The molecular dynamics simulations reported above describe the properties of flat mixed monolayers, pulled by forces normal to the plane of the interface. We have produced an essentially two dimensional model of surface tension *γ*_MD_(APL), that cannot, as such, predict the evolving dynamics of a spherical interface under negative pressure. An approach is required which extends the Laplace equation (2) out of equilibrium and allows bubble radii to vary over time, *r*(*t*), as a result of imbalances in the forces acing on their lipid coatings.^12^

In our previous work,^16^ we showed that a nanobubble under tension sits in a potential well between two maxima: one occurs at the critical radius with respect to dissolution (*r* → 0) and the other at the critical radius to embolism (*r* → ∞). The stationary points can be identified by differentiating the Gibbs free energy of a bubble with respect to radius, to produce5
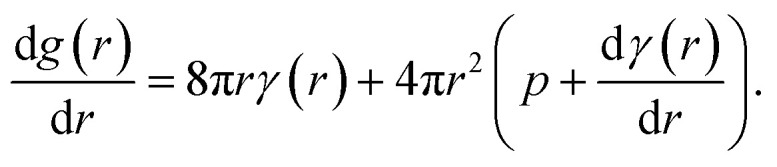


The first critical radius can be identified by assuming it takes place at a size where the surface tension is static rather than dynamic, *i.e.* d*γ*(*r*)/d*r* = 0. In this limit, the Laplace equation (2) is immediately recovered, as is the well-known Classical Nucleation Theory activation barrier height for cavitation of a void within liquid water,^37^6
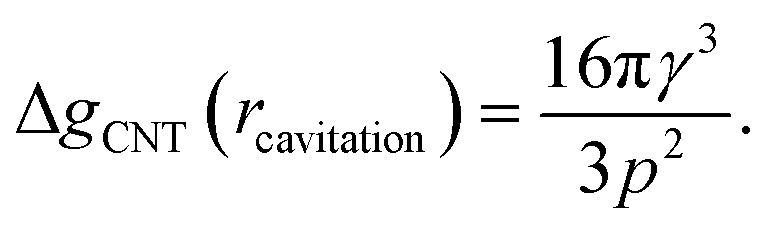


Conversely, the equilibrium radius and the critical radius with respect to embolism both occur in the regime where surface tension is dynamic, meaning they are solutions to the full differential equation (5). The time dependent size of a bubble subject to this potential energy surface should then follow the modified Rayleigh–Plesset equation of motion,^17,18^ sometimes referred to as the Rayleigh, Plesset, Noltingk, Neppiras, and Poritsky equation:^41^7
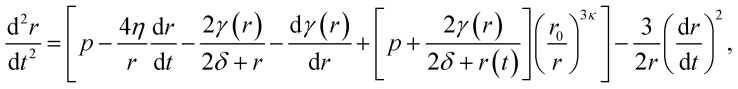
where *η* is the viscosity of water, *κ* is the polytropic index of the gas within the bubble, and *δ* is the Tolman length, a measure of the curvature dependence of *γ*. The value of *κ* was set as 1.4, representing slightly non-isothermal expansion. There is little consistency in the literature as to the sign *δ* should adopt, but it is generally considered to be sub-nm or sub-Å in magnitude,^42^ meaning the correction will be minimal at the *r* range studied here. A literature review revealed that different values of *δ* have been calculated for nucleating bubbles,^43^ lipid monolayers^44^ and water under negative pressure.^3^ Here we have used a positive value, one tenth the Lennard-Jones diameter of our water model, *i.e. δ* = +0.0316 nm, proposed for bubbles under negative pressure by Kashchiev.^45^

**Fig. 7:**
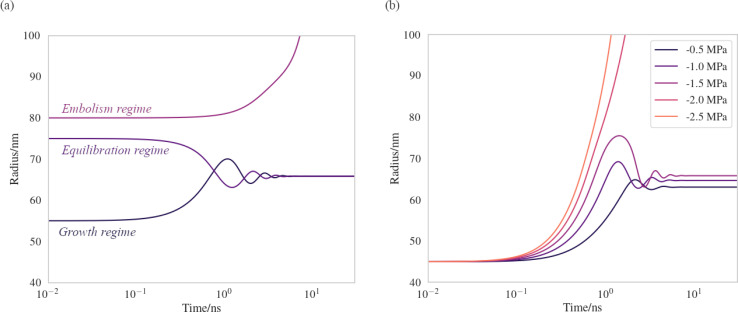
Bubble dynamics produced by integration of the Rayleigh–Plesset equation (eqn (7)), assuming *T* = 270 K, *N*_lipids_ = 10^5^ and a dynamic surface tension *γ*(*r*). (a) *p* = −1.5 MPa, with varying starting radii, *r*_0_. (b) *r*_0_ = 45 nm, with varying external negative pressure *p*. Further details in main text.

We have produced trajectories by numerically integrating equation (7), in the doubly metastable supercooled (*T* = 270 K) + negative pressure (*p* = −1.5 MPa) state, with the number of lipids in the bubble coating, *N*_lipids_ = 10^5^. The large number was used to bring the equilibrium radius, *r*_eq_, in line with that observed with nanoparticle tracking analysis of xylem nanobubbles by Guan *et al.*,^46^ namely between 50 and 100 nm. The *γ*_MD_ values were smoothed to produce *γ*(*r*) using a sum of two error functions (Fig. S4†). The dynamics were found to conform to three broad regimes depending on the “injection radius”, *r*_0_, of the bubbles. Example dynamics in the three regimes, which we call growth, equilibration and embolism, are presented in Fig. 7a.

Bubbles injected below their equilibrium radius *r*_eq_ exhibit *γ*(*r*) values close to zero and so d*r*/d*t* is strongly positive, leading to growth until the forces balance. Damped oscillations in size are observed on the order of nanoseconds, as the surface over- and undershoots its equilibrium size, with kinetic energy being dissipated by the viscosity each time. Between *r*_eq_ and a second turning point, which we call *r*_crit_, *γ*(*r*) is highly dynamic and so d*γ*(*r*)/d*r* is maximised in this regime. A disequilibrium exists where the inward forces acting on the interface dominate the outward, and bubble size is spontaneously reduced (d*r*/d*t* < 0). The activation barriers to embolism and dissolution are essentially insurmountable and the bubble size once again fluctuates towards a stable value.

Above *r*_crit_, the volumetric work term *p*(*r*_0_/*r*)^3*κ*^ is maximised and d*γ*(*r*)/d*r* is small, as this regime corresponds to areas per lipid within the monolayer coating above which the surface pressure is close to zero. As such, d*r*/d*t* becomes positive once again. We note that *r*_crit_ is strictly the critical radius with respect to embolism and is therefore separate from *r*_cavitation_, and only arises because d*γ*(*r*)/d*r* ≠ 0. By fixing the starting radius and decreasing the applied negative pressure from *p* = −0.5 MPa to −2.5 MPa (Fig. 7b) it is possible to observe the bubble dynamics becoming progressively less stable, as *r*_crit_ decreases. In the limit of extreme negative pressure (*p* = −2.0 and −2.5 MPa), no equilibration regime is observed and all bubbles above *r*_cavitation_ embolise, as the volumetric work dominates the dynamics.

Bubble dynamics were also integrated at *T* = 310 K, and broadly similar behaviour was observed in all three regimes, albeit with more rapid, less damped oscillations (Fig. S3†). As there is no representation of gas or lipid diffusion towards the expanding interface in this model, or any kinetic limitation this might impose on bubble growth or shrinkage, the timescales over which these dynamics manifest are largely arbitrary and not representative of a chemically realistic system. We consider this a sensitivity study and the trajectories indicative of the ‘fastest possible’ direction of travel across the potential energy surface (eqn (5)) the bubble experiences.

## 4 Conclusion

The role played by nanobubbles in the plant hydraulic system is beginning to come into sharper focus. Predicting under which physical conditions they will expand into embolism is of extreme importance, and requires a rigorous understanding of their surface coatings. By producing pressure:area isotherms of a biologically relevant lipid monolayer, we have shown that the range of surface tensions possible at both temperate and undercooled conditions varies smoothly from zero to that of water. The occurrence of the LC phase within the lipid tail groups was found to be initially low (*ca.* 22%) due to the polyunsaturated nature of plant lipid tails, and reduced monotonically as the monolayer was pulled apart under tension. Once the lipid tails had fully expanded into the LE phase, pore formation, followed by rupture, occurred.

A more negative pressure was required to induce rupture in the undercooled monolayers, a result that is consistent with observations of hydraulic stability in xylem conduits. Since pore formation is an activated process, the barrier height to embolising a nanobubble should be proportional to the difference in surface tension between the equilibrium and ruptured states of the monolayer coating. Therefore, reduced surface pressure, in combination with a more condensed equilibrium area per lipid, will likely stabilize bubbles at lower temperatures.

Rayleigh–Plesset simulations on the tens of nanosecond timescale show that the equilibrium radius of a bubble is largely determined by the number of lipids present in its coating. Bubbles formed above their equilibrium radius (*i.e.* with an expanded monolayer) were found to sometimes embolise immediately, assuming they were injected with a mean area per lipid exceeding a critical value. Bubbles whose starting radius was between *r*_cavitation_ and *r*_crit_ were found to be capable of equilibrating to a stable size, provided the pressure within the surrounding liquid was less negative than −2.0 MPa.

As such, this method provides a useful theoretical tool to estimate critical radii with respect to embolism, as well as the response of bubble size to changes in the external pressure it experiences. A more complex model containing additional physics, such as Brownian motion or gas diffusion towards the bubble interface, could be used to simulate dynamics at a timescale closer to that observable by experiment, as well as to probe the effect of temperature on the equilibrium between dissolved gas and vapour within xylem sap. It may also be desirable to investigate the surface properties of other relevant lipids, as well as non-lipid surfactant classes present in plants, such as triacylglycerides^12^ or denatured proteins.^47^

## Author contributions

Simulations and analysis were conducted by SI under the supervision of TV and HV. BR provided methodological expertise. Manuscript was written by SI and BR.

## Conflicts of interest

There are no conflicts to declare.

## Supplementary Material

NA-006-D4NA00316K-s001
