# Supplementary material for: Ruptures of mixed lipid monolayers under tension and supercooling: implications for nanobubbles in plants
Source: Nanoscale Adv. 2024 Jun 11;6(15):3775–84. doi: 10.1039/d4na00316k (PMC11265596; doi:10.1039/d4na00316k)
Supplement: NA-006-D4NA00316K-s001 [file NA-006-D4NA00316K-s001.pdf]

Electronic Supplementary Material (ESI) for Nanoscale Advances.
This journal is © The Royal Society of Chemistry 2024

Supplementary Information for: Ruptures of Mixed Lipid Monolayers Under Tension and Supercooling: Implications for Nanobubbles in Plants

Stephen Ingram[a], Bernhard Reischl[a], Timo Vesala[a,b] and Hanna Vehkamäki[a]

[a]Institute for Atmospheric and Earth System Research/Physics,
Faculty of Science, P.O. Box 64,
[b]Institute for Atmospheric and Earth System Research/Forest Sciences,
Faculty of Agriculture and Forestry, P.O. Box 27,
University of Helsinki,
Helsinki, FI-00014 Finland

stephen.ingram@helsinki.fi
hanna.vehkamaki@helsinki.fi

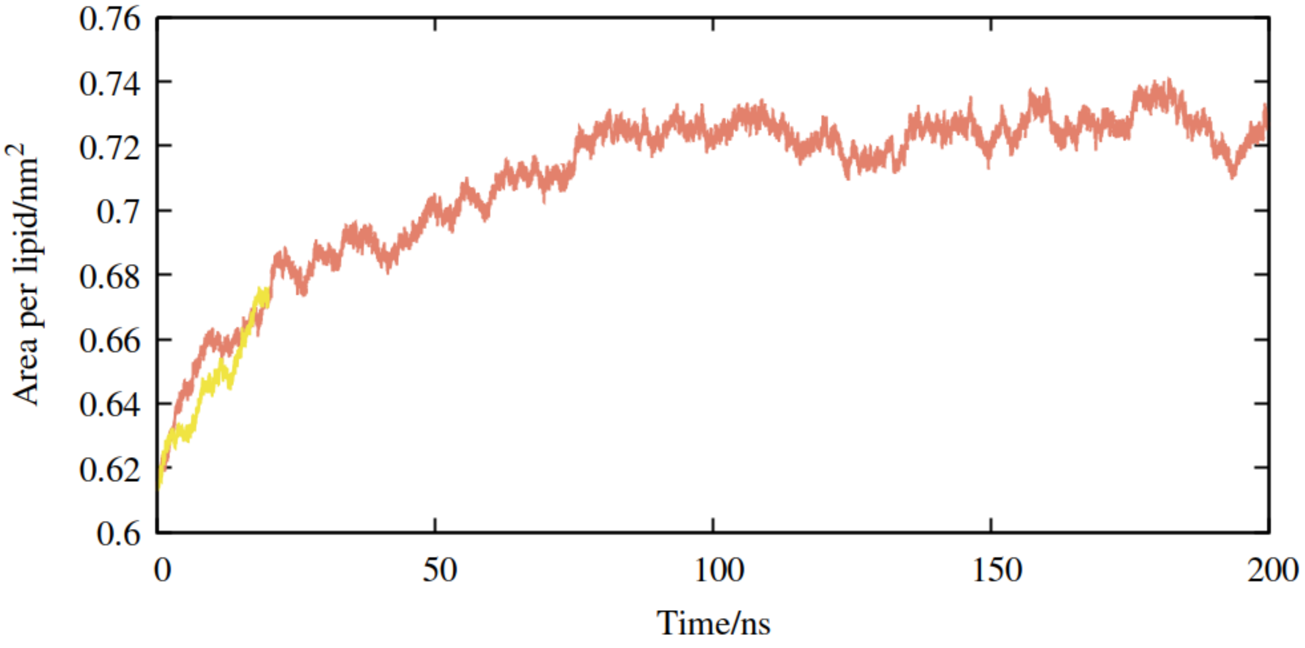

Figure S1: Expansion of the mixed monolayer at $T = 270$ K and $p = -2.5$ MPa. Two simulations are presented, with the same starting coordinates, but differing random seeds for velocity generation. Yellow data is the same as that presented in the second trajectory of Figure 3 in the main text.

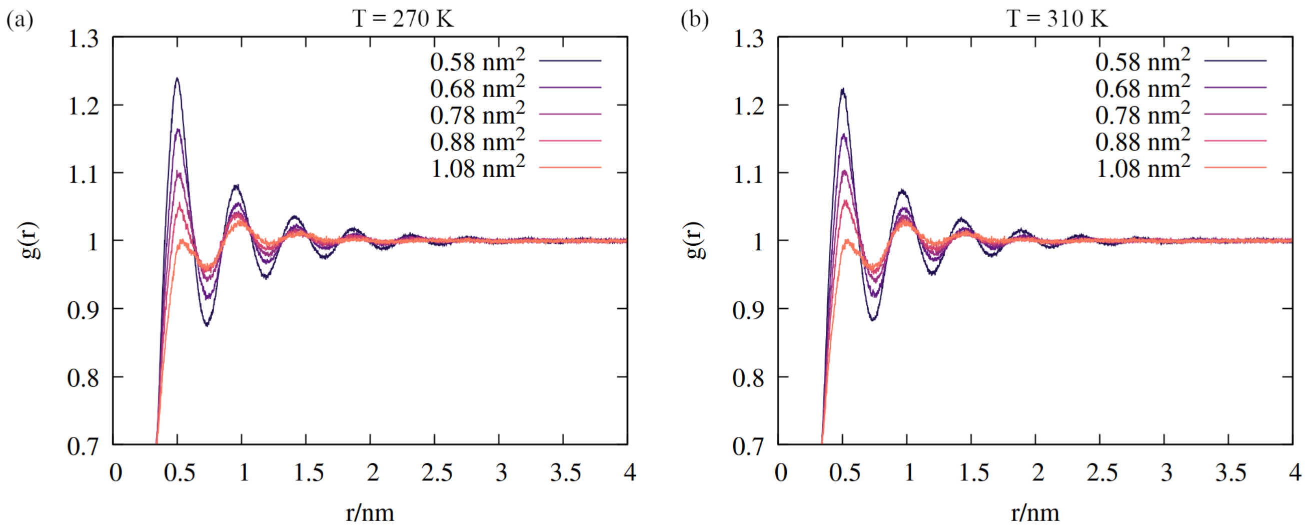

Figure S2: Two dimensional radial distribution functions (RDFs) of lipid tail group atoms, at increasing areas per lipid. (a) $T = 270$ K and (b) $T = 270$ K. RDFs were calculated using the same atomic coordinates as those presented in Figure 4 in the main text, output every 10 ps between $t = 10$ and 60 ns.

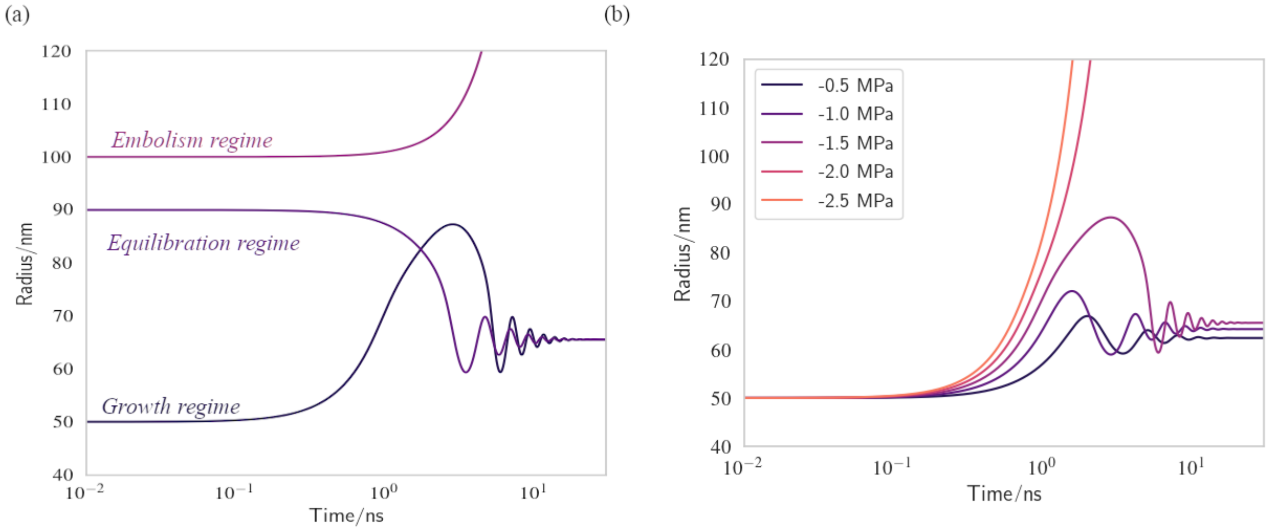

Figure S3: Bubble dynamics produced by integration of the Rayleigh-Plesset equation (Eq. 7), assuming $T = 310$ K, $N_{\text{lipids}} = 10^5$, and a dynamic surface tension $\gamma(r)$. (a) $p = -1.5$ MPa, with varying starting radii, r_0. (b) r_0=50 nm, with varying external negative pressure, p. Further details in main text and Methods.

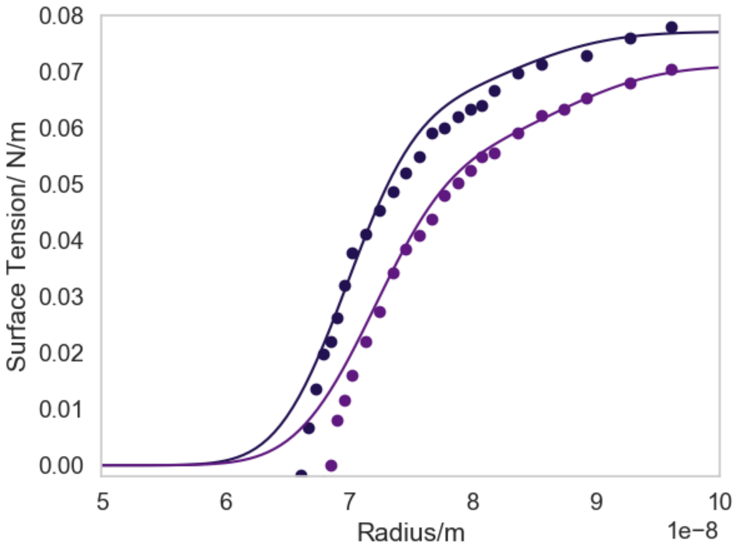

Figure S4: Smoothed parameterisations of the calculated surface tensions, $\gamma_{\text{MD}}(T)$, as a function of radius, r, where $r = \sqrt{(APL \cdot N_{\text{Lipids}})/4\pi}$. Purple points and lines are at $T = 310$ K, black are at $T = 270$ K.
